# Bacterial characteristics of carbapenem-resistant *Enterobacteriaceae* (CRE) colonized strains and their correlation with subsequent infection

**DOI:** 10.1186/s12879-021-06315-0

**Published:** 2021-07-02

**Authors:** Qun Lin, Yue Wang, Jing Yu, Shusheng Li, Yicheng Zhang, Hui Wang, Xiaoquan Lai, Dong Liu, Liyan Mao, Ying Luo, Guoxing Tang, Zhongju Chen, Ziyong Sun

**Affiliations:** 1 Department of Laboratory Medicine, Tongji Hospital, Tongji Medical College, Huazhong University of Science and Technology, Wuhan, China; 2 The Third Affiliated Hospital of Zhengzhou University, Zhengzhou, China; 3 Department of Emergency Medicine, Tongji Hospital, Tongji Medical College, Huazhong University of Science and Technology, Wuhan, China; 4 Department of Haematology, Tongji Hospital, Tongji Medical College, Huazhong University of Science and Technology, Wuhan, China; 5 Department of Nursing, Tongji Hospital, Tongji Medical College, Huazhong University of Science and Technology, Wuhan, China; 6 Department of Nosocomial Infection Management, Tongji Hospital, Tongji Medical College, Huazhong University of Science and Technology, Wuhan, China; 7 Department of Pharmacy, Tongji Hospital, TongjiMedical College, Huazhong University of Science and Technology, Wuhan, China

**Keywords:** Carbapenem-resistant *Enterobacteriaceae*, Intestinal colonization, Risk factor, Bacterial characteristic

## Abstract

**Background:**

Searching the risk factors for carbapenem-resistant *Enterobacteriaceae* (CRE) infection is important in clinical practice. In the present study, we aim to investigate bacterial characteristics of colonizing strains and their correlation with subsequent CRE infection.

**Methods:**

Between May 2018 and January 2019, patients hospitalized in the department of haematology and intensive care unit (ICU) were screened for CRE by rectal swabs and monitored for the outcome of infection. We identified the species and carbapenemase-encoding genes of colonizing strains and performed antimicrobial susceptibility tests and multilocus sequence typing (MLST). Risk factors for subsequent CRE infections were ascertained by univariate and multivariable analysis.

**Results:**

We collected a total of 219 colonizing strains from 153 patients. *Klebsiella pneumoniae* was the most abundant species, and MLST analysis showed rich diversity. *K. pneumoniae* carbapenemase (KPC) was predominant in the infection group (72.4%). In the non-infection group, 35.4% of strains were non-carbapenemase-producing CRE (NCP-CRE), and New Delhi metallo-β-lactamase (NDM) was predominant (42.2%). The rate of high-level carbapenem resistance (minimum inhibitory concentration [MIC] ≥ 64 mg/L for meropenem and ertapenem, ≥ 32 mg/L for imipenem) was remarkably higher in the infection group than in the non-infection group (*P* <  0.001). Univariate analysis showed that *K. pneumoniae*, high-level carbapenem resistance, CP-CRE and KPC-CRE were infection risk factors after CRE colonization. On multivariable analysis with different carbapenemase dichotomizations, KPC-CRE (adjusted odds ratio [aOR], 4.507; 95% confidence interval [CI], 1.339–15.171; *P* = 0.015) or imipenem MIC ≥ 32 mg/L (aOR, 9.515; 95% CI, 1.617–55.977; *P* = 0.013) were respectively identified as independent risk factors for subsequent infection.

**Conclusions:**

Patients colonized with KPC-CRE or strains with an imipenem MIC ≥ 32 mg/L were at particularly high risk of subsequent CRE infections during their hospital stay.

**Supplementary Information:**

The online version contains supplementary material available at 10.1186/s12879-021-06315-0.

## Background

Carbapenem-resistant *Enterobacteriaceae* (CRE) infections are of major concern to clinicians and public health authorities due to increasing prevalence, rapid regional dissemination, limited therapeutic options and deleterious patient outcomes (mortality rates are 40 ± 10%) [1]. CRE carriage is responsible for the incidence of clinical infection [2–6], and it has been reported that colonization with CRE was associated with at least a two-fold increased risk of infection by the colonizing strain [3]. Many guidelines for the prevention and control of these organisms have been developed by health organizations, including the Centers for Disease Control and Prevention (CDC), the European Centre for Disease Prevention and Control, and the World Health Organization [7–9]. A series of clinical reports have shown that individually or nationally directed infection control interventions can effectively reduce CRE transmission and infection rates [4–6]. Moreover, researchers have increasingly explored strategies to decolonize CRE to interrupt pathways between colonization and subsequent infections [10–13]. Despite remarkable effects, there are various challenges to implement these interventions or strategies [14]. Searching CRE colonization patients who are at high risk of infection is an urgent priority, as it can guide us whether additional interventions are needed and limit decolonization strategy use.

Although many studies have been performed to identify risk factors for clinical CRE infections, which highlighted the analysis of clinical data of patients [3, 15–18], few have explored bacterial characteristics of colonizing strains and their correlation with subsequent CRE infection. It was reported that patients colonized with carbapenemase-producing CRE (CP-CRE) were more likely than non-carbapenemase-producing CRE (NCP-CRE)-colonized patients to develop CRE infections during hospitalization [19]. Previous studies illustrated that some characteristics of infection strains, namely, carbapenemase-encoding genes and minimum inhibitory concentration (MIC) values of carbapenem, were closely related to the outcome of CRE-infected patients [20]. Therefore, we postulated that some microbiological parameters of CRE-colonizing isolates may be risk factors of subsequent infections in patients colonized with CRE.

The study were conducted among patients hospitalized in department of haematology and intensive care unit (ICU), which are at particularly high risk of infecting CRE during their hospital stay as compromised immune systems, lengthy unit stays, and significant rates of device and antibiotic utilization [19]. We identified the microbiological parameters of colonizing isolates, including genus and species, phenotypic carbapenem resistance profile, carbapenemase production status, carbapenemase-encoding genes and multilocus sequence typing (MLST). Subsequently, univariate and multivariable analyses were conducted to find their correlation with subsequent CRE infection. We found that imipenem MIC ≥ 32 mg/L or *Klebsiella pneumoniae* carbapenemase (KPC)-positive CRE-colonized patients were at high risk of subsequent CRE infections during their hospital stay.

## Materials and methods

### Research setting and ethics statement

From May 2018 to January 2019, we carried out this study at Tongji Hospital, the largest hospital in the region of central China. In the department of haematology and ICU, we initiated a coordinated and comprehensive intervention of CRE, which mainly included implementation of CRE screening, contact precautions, patient isolation, and antibiotic management [7–9, 21]. All inpatients (including transferred and re-admitted patients) were routinely screened for CRE by rectal swabs on admission and twice a week thereafter until discharge or infection. For CRE-colonized and -infected patients, isolation precautions were applied according to CDC guidelines, and removed if they developed decolonization [7]. Decolonization was defined as two consecutive negative cultures within 48 h, and the second sample was confirmed to be carbapenemase-negative by the Xpert® Carba-R Assay (Cepheid, Sunnyvale, California).

We collected all CRE-colonizing strains from adult patients (≥ 18 years old), and divided them into infection and non-infection groups, according to whether the colonized patient had a subsequent CRE infection. The infection group included all CRE colonizing isolates obtained from patients who subsequently had CRE infections. Strains isolated from colonized patients who did not develop CRE infections during their hospital stay were subsumed by the non-infected group.

This study was approved by the ethical committee of Tongji hospital, Tongji Medical College, Huazhong University of Science and Technology, Wuhan, China.

### Bacterial isolate collection and identification

Rectal swabs were consecutively obtained from patients and screened for CRE with selective chromogenic agar (Zhengzhou Dianshi biotechnology Co., Ltd., China). Cultured isolates were identified by matrix-assisted laser desorption/ionization time-of-flight mass spectrometry (MALDI-TOF MS, Bruker Daltonics Inc., Billerica, Massachusetts), and then carbapenem (meropenem and imipenem) antimicrobial susceptibility testing was performed to confirm CRE by the disk diffusion method [22]. *Enterobacteriaceae* that were resistant to meropenem or imipenem were classified as CRE.

### Antibiotic susceptibility testing

According to the Clinical and Laboratory Standards Institute (CLSI) guidelines [22], we performed antibiotic susceptibility testing using the broth microdilution method to determine MICs of cefepime, cefoxitin, ceftazidime, aztreonam, ertapenem, imipenem, meropenem, gentamicin, amikacin, minocycline, ciprofloxacin, fosfomycin, piperacillin-tazobactam, trimethoprim-sulfamethoxazole, colistin and tigecycline. All antibiotics, except tigecycline and colistin, were interpreted according to the standard of the CLSI document. For tigecycline and colistin, the European Committee on Antimicrobial Susceptibility Testing (EUCAST) breakpoint was used. *Escherichia coli* ATCC 25922 and *Pseudomonas aeruginosa* ATCC27853 were used as quality control standards.

### Investigation of resistance mechanisms

For all CRE strains, the modified carbapenem inactivation method (mCIM) was conducted to identify carbapenemase production [23]. For CP-CRE strains, polymerase chain reaction (PCR) was performed to detect five common carbapenemase-encoding genes, including *bla*_KPC_, *bla*_IMP_, *bla*_VIM_, *bla*_NDM_ and *bla*_OXA-48_ [24]. CP-CRE strains without common genes were further tested uncommon carbapenemase-encoding genes, including *bla*_GES_, *bla*_VEB_, *bla*_PER_, *bla*_SME_ and *bla*_IMI_ [25–28]. The PCR products were sequenced and analysed using BLAST (http://www.ncbi.nlm.nih.gov/BLAST).

### Multilocus sequence typing

Multilocus sequence typing (MLST) of *K. pneumoniae* was performed following the protocol described on the Pasteur Institute MLST website (http://www.pasteur.fr/recherche/genopole/PF8/mlst/Kpneumoniae.html). The sequences of seven housekeeping genes and sequence types (STs) were assigned using online MLST databases.

### Statistical analysis

Data were analysed using SPSS v.19.0 software (SPSS Inc., Chicago, IL, USA). MICs were analysed both as ordinal and as dichotomized variables. We calculated the Youden index (sensitivity + specificity – 1) at each possible cutoff value for dichotomized MICs. The significant differences between different groups were analysed using the chi-square (χ^2^) test or Fisher’s exact test, as appropriate. Univariate logistic regression analyses were carried out to assess the relevant risk factors of CRE infection. Only significantly different factors were subsequently included in multivariable analyses, which were constructed using stepwise model selection and manually curated. Statistical significance was determined as *P* <  0.05. Because carbapenemase can be distinguished by production status and different carbapenemase-encoding genes, we conducted multivariable analyses twice. Odds ratio (OR) with 95% confidence interval (CI) was presented for the logistic regression analysis.

## Results

### Study population and distribution of CRE colonizing isolates

A total of 219 CRE colonizing strains were collected from 153 patients, of whom 29 individuals developed CRE infections during hospitalization (Supplementary Fig. 1). In the infection group, we obtained 23 colonizing isolates from the ICU and 35 isolates from the department of haematology. *K. pneumoniae* was the most abundant species (81.0%), followed by *E. coli* (12.1%). In the non-infection group, there were 161 strains and 70.2% of strains were from the department of haematology. Thereinto, *K. pneumoniae* accounted for 53.4% and *E. coli* accounted for 29.2%. The two groups differed remarkably in terms of bacterial species (*P* = 0.001), and there were no obvious differences in medical department proportions (*P =* 0.170) (Table 1).

**Table 1:** Characteristics of carbapenem-resistant *Enterobacteriaceae* (CRE)-colonizing strains in different groups

Variables	No. (%) of isolates		
	Infection group (*n* = 58)	Non-infection group (*n* = 161)	*P*
**Medical department**			
Intensive care unit	23 (39.7)	48 (29.8)	0.170
Department of haematology	35 (60.3)	113 (70.2)	
**Species**			
*Klebsiella pneumoniae*	47 (81.0)	86 (53.4)	< 0.001
ST11	42 (89.4)	26 (30.2)	< 0.001
ST37	1 (2.1)	10 (11.6)	0.116
Other ST	4 (8.5)	50 (58.1)	< 0.001
*Escherichia coli*	7 (12.1)	47 (29.2)	0.009
Other CRE^a^	4 (6.9)	28 (17.4)	0.085
**MIC of meropenem**			
Susceptible	1 (1.7)	15 (9.3)	0.107
Intermediate	4 (6.9)	12 (7.5)	> 0.999
Resistant	53 (91.4)	134 (83.2)	0.132
**MIC of imipenem**			
Susceptible	5 (8.6)	31 (19.3)	0.061
Intermediate	2 (3.5)	18 (11.2)	0.137
Resistant	51 (87.9)	112 (69.6)	0.006
**MIC of ertapenem**			
Susceptible	1 (1.7)	0	0.265
Intermediate	0	0	
Resistant	57 (98.3)	161 (100)	0.265
**Carbapenemase**			
Positive	51	104	
*bla*_KPC-2_	42 (72.4)	27 (16.8)	< 0.001
*bla*_NDM_^b^	9 (15.5)	68 (42.2)	< 0.001
Other^c^	0	9 (5.6)	0.116
Negative	7 (12.07)	57 (35.4)	0.001

Abbreviations: *CRE* carbapenem-resistant *Enterobacteriaceae*; *MIC* minimum inhibitory concentration

Note:

a Four other CRE in the infection group was *Enterobacter cloacae* (*n* = 2), *Enterobacter kobei* (*n* = 1) and *Morganella morganii* (*n* = 1); 28 other CRE in the Non-infection group was *Citrobacter amalonaticus* (*n* = 1), *Citrobacter freundii* (*n* = 11), *E. cloacae* (*n* = 8), *E. kobei* (*n* = 3), *Klebsiella oxytoca* (*n* = 2), *Raoultella ornithinolytica* (*n* = 2) and *Leclercia adecarboxylata* (*n* = 1).

b Nine strains with *bla*_NDM_ in the infection group was *bla*_NDM-1_ (*n* = 4) and *bla*_NDM-5_ (*n* = 5); 68 strains with *bla*_NDM_ in the Non-infection group was *bla*_NDM-1_ (*n* = 30), *bla*_NDM-4_ (*n* = 1), *bla*_NDM-5_ (*n* = 35), and *bla*_NDM-7_ (*n* = 2).

c Including two strains co-harbouring *bla*_KPC-2_ and *bla*_NDM-1_ (*K. pneumoniae* and *K. oxytoca*, *n* = 1), two *E. coli* with *bla*_VIM-1_, two strains with *bla*_IMP-4_ (*K. pneumoniae* and *R. ornithinolytica*, *n* = 1), one *R. ornithinolytica* with *bla*_oxa-48_ and two strains which didn’t harbour the tested carbapenemase-encoding genes (*E. cloacae* and *K. pneumoniae*, *n* = 1).

### Characteristics of *K. pneumoniae* STs

A total of 36 distinct STs were identified among 133 carbapenem-resistant *K. pneumoniae* (CR-KP) (Supplementary Table 1). As depicted in Table 1, ST11 was the most prevalent ST in the infection group (89.4%). In the non-infection group, a total of 33 STs were identified, among which ST11 was the most common type (30.2%), followed by ST37 (11.6%), ST15 (8.1%) and ST147 (7.0%). A univariable analysis showed a difference in the proportion of STs between the two groups (*P* <  0.001).

### Screening for carbapenemase-encoding genes

From all strains, 155 (70.8%) were found to produce carbapenemases (Table 1). The major carbapenemase-encoding genes were KPC-type (*n* = 69) and New Delhi metallo-β-lactamase (NDM)-type (*n* = 77). All detected KPC-type genes were *bla*_KPC-2_, while NDM-type genes included *bla*_NDM-1_ (*n* = 34), *bla*_NDM-4_ (*n* = 1), *bla*_NDM-5_ (*n* = 40), and *bla*_NDM-7_ (*n* = 2). Other common carbapenemase-encoding genes, namely *bla*_IMP-4_ (*n* = 2), *bla*_VIM-1_ (*n* = 2) and *bla*_OXA-48_ (*n* = 1), were also found. Uncommon genes were not detected. Two strains co-harbouring *bla*_KPC-2_ and *bla*_NDM-1_ and two CP-CRE strains which didn’t harbour the tested genes were found.

In the infection group, 87.9% of strains were CP-CRE and KPC (72.4%) was the most common carbapenemase type. In the non-infection group, approximately 64.6% of strains were CP-CRE, among which NDM (42.2%) was the most abundant, followed by KPC (16.8%). There were noticeable differences in carbapenemases between the two groups (*P* <  0.001). The KPC production status was remarkably associated with CRE infection after intestinal CRE colonization.

### Antimicrobial susceptibility testing results

The antimicrobial susceptibility of CRE colonizing isolates is shown in Table 2. In total, rectal CRE strains showed high susceptibility to colistin (92.2%), followed by tigecycline (83.1%). Compared with *K. pneumonia*, *E. coli* was more susceptible to gentamicin (59.3% vs. 21.1%), amikacin (87.0% vs. 45.1%) and fosfomycin (74.1% vs. 11.3%). NDM-positive strains were more susceptible to aztreonam (37.7% vs. 0), gentamicin (46.8% vs. 7.2%), amikacin (87.0% vs. 14.5%) and fosfomycin (63.6% vs. 0) than KPC-producing strains. NCP-CRE was more resistant to tigecycline and trimethoprim-sulfamethoxazole than CP-CRE. The infection and non-infection groups differed significantly in terms of susceptibility to gentamicin (15.5% vs. 37.9%), amikacin (31.0% vs. 71.4%), fosfomycin (6.9% vs. 41.6%), tigecycline (93.1% vs. 79.5%) and trimethoprim-sulfamethoxazole (48.3% vs. 19.3%).

**Table 2:** Antimicrobial susceptibility testing results of carbapenem-resistant *Enterobacteriaceae* (CRE)-colonizing strains

Antibiotics	All Strains (*n* = 219)		Non-infection group (*n* = 161)		Infection group (*n* = 58)		*Klebsiella pneumonia* (*n* = 133)		*Escherichia coli* (*n* = 54)		Other CRE (*n* = 32)		KPC (*n* = 69)		NDM (*n* = 77)		Other (*n* = 9)		NCP-CRE (*n* = 64)	
	R%	S%	R%	S%	R%	S%	R%	S%	R%	S%	R%	S%	R%	S%	R%	S%	R%	S%	R%	S%
Cefepime	96.8	1.4	96.3	1.2	98.3	1.7	96.2	1.5	98.2	0	96.9	3.1	100	0	100	0	100	0	89.1	4.7
Cefoxitin	97.3	0.5	97.5	0	96.6	1.7	96.2	0	100	0	96.9	3.1	95.7	0	100	0	100	0	95.3	1.6
Ceftazidime	97.7	1.4	97.5	1.2	98.3	1.7	97.7	0.8	98.2	1.9	96.9	3.1	100	0	100	0	100	0	92.2	4.7
Aztreonam	81.3	16.4	78.9	18.0	87.9	12.1	91.0	7.5	66.7	31.5	65.6	28.1	100	0	58.4	37.7	55.6	22.2	92.2	7.8
Ertapenem	99.5	0.5	100	0	98.3	1.7	100	0	100	0	96.9	3.1	100	0	100	0	100	0	98.4	1.6
Imipenem	74.4	16.4	69.6	19.3	87.9	8.6	72.2	17.3	72.2	22.2	87.5	3.1	100	0	100	0	66.7	11.1	17.2	54.7
Meropenem	85.4	7.3	83.2	9.3	91.4	1.7	85.0	4.5	81.5	14.8	93.8	6.3	100	0	100	0	100	0	50.0	25.0
Gentamicin	67.6	32.0	61.5	37.9	84.5	15.5	78.2	21.1	40.7	59.3	68.8	31.3	92.8	7.2	51.9	46.8	55.6	44.4	60.9	39.1
Amikacin	37.9	60.7	26.7	71.4	69.0	31.0	54.1	45.1	11.1	87.0	15.6	81.3	85.5	14.5	13.0	87.0	22.2	77.8	18.8	76.6
Minocycline	42.5	44.8	46.6	42.9	31.0	50.0	45.9	41.4	35.2	55.6	40.6	40.6	23.2	60.9	40.3	48.1	0	88.9	71.9	17.2
Ciprofloxacin	86.3	11.0	84.5	12.4	91.4	6.9	90.2	7.5	87.0	9.3	68.8	28.1	100	0	75.3	20.8	55.6	22.2	89.1	9.4
Fosfomycin	57.1	32.4	46.6	41.6	86.2	6.9	78.2	11.3	22.2	74.1	28.1	50.0	100	0	18.2	63.6	33.3	66.7	60.9	25.0
Piperacillin-tazobactam	95.4	3.2	95.0	3.7	96.6	1.7	94.7	3.0	98.2	1.9	93.8	6.3	100	0	100	0	88.9	11.1	85.9	9.4
Trimethoprim-sulfamethoxazole	73.1	26.9	80.8	19.3	51.7	48.3	68.4	31.6	77.8	22.2	84.4	15.6	47.8	52.2	84.4	15.6	44.4	55.6	90.6	9.4
Colistin	7.8	92.2	9.9	90.1	1.7	98.3	6.0	94.0	3.7	96.3	21.9	78.1	2.9	97.1	10.4	89.6	0	100	9.4	90.6
Tigecycline	16.9	83.1	20.5	79.5	6.9	93.1	26.3	73.7	0	100	6.3	93.8	7.2	92.8	10.4	89.6	0	100	37.5	62.5

Abbreviations: *R* resistant; *S* susceptible; *CRE* carbapenem-resistant *Enterobacteriaceae*; *KPC K. pneumoniae* carbapenemase; *NDM* New Delhi metallo-β-lactamase; *NCP-CRE* non-carbapenemase-producing CRE

### Evaluation of cutoff values for dichotomized carbapenem MICs

**Fig. 1:**
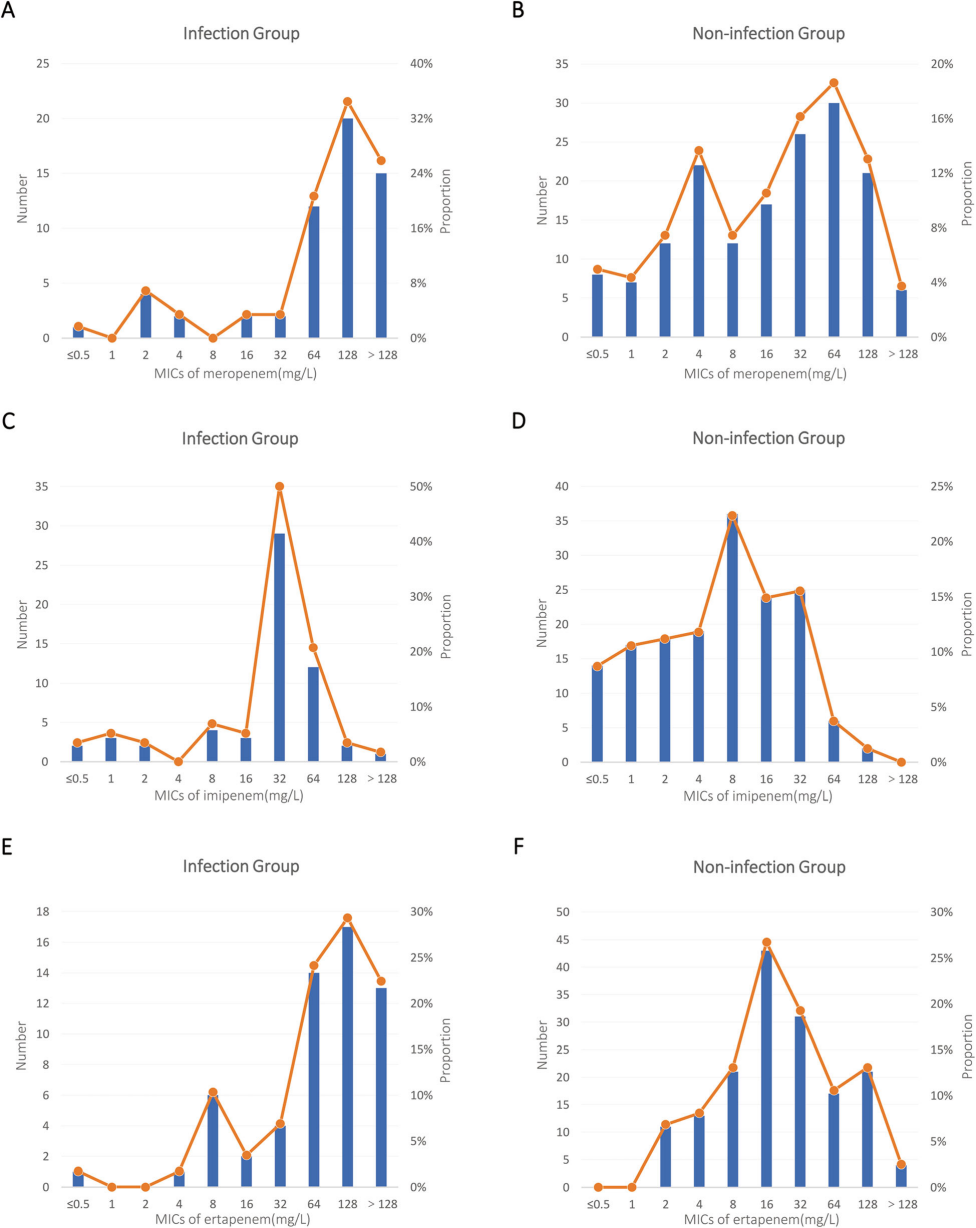
The distribution of minimum inhibitory concentrations (MICs) of carbapenem (meropenem, imipenem and ertapenem) in different groups of carbapenem-resistant *Enterobacteriaceae* colonizing strains. **A** Meropenem MIC distribution in the infection group (*n* = 58). **B** Meropenem MIC distribution in the non-infection group (*n* = 161). **C** Imipenem MIC distribution in the infection group. **D** Imipenem MIC distribution in the non-infection group. (**E**) Ertapenem MIC distribution in the infection group. (**F**) Ertapenem MIC distribution in the non-infection group

According to carbapenem breakpoints, there were no obvious differences between the two groups except for the rate of resistance to imipenem (Table 1). The distributions of MICs of carbapenem in the two groups are shown in Fig. 1. In the infection group, there was only one peak in every carbapenem antibiotic and the peak value was high. However, the distribution in the non-infection group was relatively gentle, and the peak value was lower. Youden index was calculated to determine the most appropriate cutoff values for dichotomized MICs (Supplementary Table 2). When MICs for meropenem and ertapenem were dichotomized at < 64 mg/L vs. ≥ 64 mg/L and MICs for imipenem were dichotomized at < 32 mg/L vs. ≥ 32 mg/L, the Youden indexes were the highest. Colonizing strains with high carbapenem MICs (MIC ≥ 64 mg/L for meropenem and ertapenem, ≥ 32 mg/L for imipenem) were risk factors for subsequent infection (*P* <  0.001).

### Relationship between different colonized bacterial factors

**Fig. 2:**
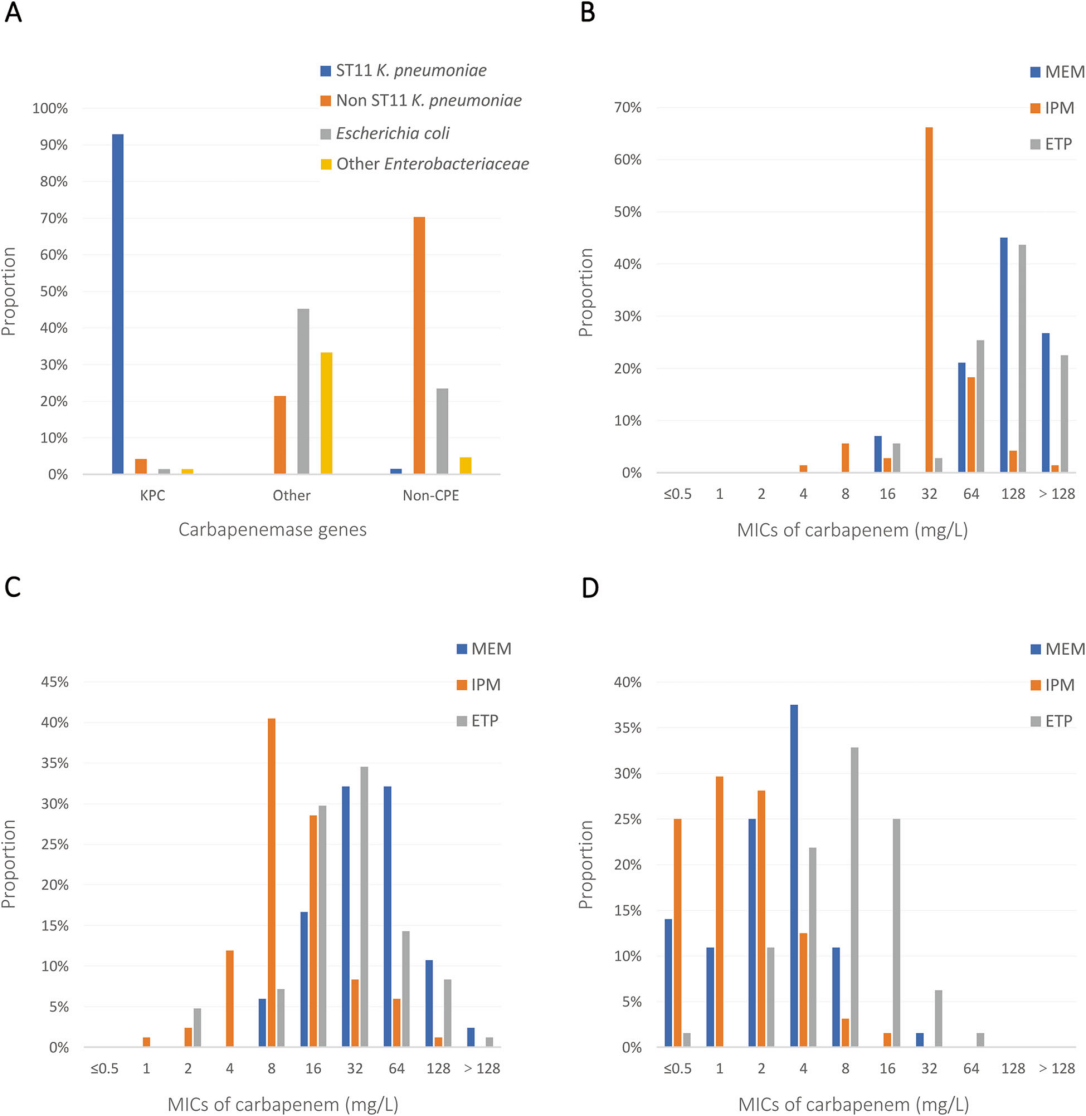
The distribution of species and carbapenem MICs among CRE-colonizing strains producing different carbapenemases. **A** Species distribution among strains producing different carbapenemases. **B** Carbapenem MICs distribution among KPC-producing strains (*n* = 71). **C** Carbapenem MIC distribution among strains producing other carbapenemases (*n* = 84). **D** Carbapenem MIC distribution among NCP-CRE strains (*n* = 64). Abbreviations: MEM, meropenem; IPM, imipenem; ETP, ertapenem; MIC, minimum inhibitory concentration; CRE, carbapenem-resistant *Enterobacteriaceae*; KPC, *K. pneumoniae* carbapenemase; NCP-CRE, non-carbapenemase-producing CRE

The distributions of species and carbapenem MICs in different carbapenemase types are shown in Fig. 2. Approximately 97.2% of KPC-positive strains were *K. pneumoniae,* among which ST11*-K. pneumoniae* (95.7%) was the most prevalent (*P* <  0.001) (Fig. 2A). The most frequently observed MICs of meropenem and ertapenem in KPC-positive strains was ≥ 64 mg/L (93.0 and 91.6%). Over 90% of KPC-CRE had high-level imipenem MICs (Fig. 2B). For strains producing other carbapenemases, the distributions of MICs focused on 16 to 128 mg/L for meropenem and ertapenem (91.7 and 87.0%), and 15.5% strains had a high level of imipenem MICs (Fig. 2C). Few NCP-CRE had high-level carbapenem MICs (Fig. 2D). In brief, rates of high-level carbapenem MICs were much higher in KPC-CRE (*P* <  0.001).

### Relationship between colonized bacterial factors and risk of CRE infection

Univariate analyses revealed that *K. pneumoniae*, meropenem MIC ≥ 64 mg/L, imipenem MIC ≥ 32 mg/L, ertapenem MIC ≥ 64 mg/L, CP-CRE and KPC-CRE were risk factors for subsequent CRE infection in CRE intestinal carriers. When combined different factors, there were no obvious improvements in the predictive ability (Table 3).

**Table 3:** Univariable analyses of bacterial factors for a subsequent infection among patients with carbapenem-resistant *Enterobacteriaceae* colonization

Variables	No. (%) of isolates		*P*	OR (95%CI)
	Infection group (*n* = 58)	Non-infection group (*n* = 161)		
**Species**				
*Klebsiella pneumoniae*	47 (81.0)	86 (53.4)	< 0.001	3.726 (1.803–7.700)
Non*-Klebsiella pneumoniae*	11 (19.0)	75 (46.6)		
**MIC of meropenem**				
< 64 mg/L	11 (19.0)	104 (64.6)	< 0.001	7.796 (3.751–16.203)
≥ 64 mg/L	47 (81.0)	57 (35.4)		
**MIC of imipenem**				
< 32 mg/L	14 (24.1)	128 (79.5)	< 0.001	12.190 (5.976–24.865)
≥ 32 mg/L	44 (75.9)	33 (20.5)		
**MIC of ertapenem**				
< 64 mg/L	14 (24.1)	119 (73.9)	< 0.001	8.905 (4.436–17.874)
≥ 64 mg/L	44 (75.9)	42 (26.1)		
**Carbapenemase**				
CP-CRE	51 (87.9)	104 (64.6)	0.001	3.993 (1.701–9.375)
NCP-CRE	7 (12.1)	57 (35.4)		
KPC-CRE	42 (72.4)	27 (16.8)	< 0.001	13.028 (6.412–26.468)
NKPC-CRE	16 (27.6)	134 (83.2)		
**Imipenem MIC & Carbapenemase**				
≥ 32 mg/L and CP-CRE	44 (75.9)	33 (20.5)	< 0.001	12.190 (5.976–24.865)
< 32 mg/L or NCP-CRE	14 (24.1)	128 (79.5)		
≥ 32 mg/L or CP-CRE	51 (87.9)	104 (64.6)	0.001	3.993 (1.701–9.375)
< 32 mg/L and NCP-CRE	7 (12.1)	57 (35.4)		
≥ 32 mg/L and KPC-CRE	40 (69.0)	24 (14.9)	< 0.001	12.685 (6.266–25.682)
< 32 mg/L or NKPC-CRE	18 (31.0)	137 (85.1)		
≥ 32 mg/L or KPC-CRE	46 (79.3)	38 (23.6)	< 0.001	12.408 (5.967–25.801)
< 32 mg/L and NKPC-CRE	12 (20.7)	123 (76.4)		

Abbreviations: *OR* adjusted odds ratio; *CI* confidence interval; *MIC* minimum inhibitory concentration; *CRE* carbapenem-resistant *Enterobacteriaceae*; *CP-CRE* carbapenemase-producing CRE; *NCP-CRE* non-carbapenemase-producing CRE; *KPC-CRE* CRE strains producing *K. pneumoniae* carbapenemase; *NKPC-CRE* CRE strains that do not produce *K. pneumoniae* carbapenemase

When dichotomizing carbapenemase by if it was KPC production or not, KPC-CRE (adjusted odds ratio [aOR], 4.507; 95% CI, 1.339–15.171; *P* = 0.015) was independently associated with a subsequent infection in a multivariable analysis. On the other hand, when dichotomizing carbapenemase by if it was CP-CRE or NCP-CRE, imipenem MIC ≥ 32 mg/L (aOR, 9.515; 95% CI, 1.617–55.977; *P* = 0.013) was the only independent factor (Supplementary Table 3).

## Discussion

CRE has been classified as an urgent threat, and CRE colonization was significantly associated with the increased risk of subsequent CRE infection [2–6]. The need to identify patients as having a high risk for CRE infection has been recognized. Many reports have evaluated risk factors for CRE infection, and some have proposed risk factor scoring models; however, these reports were focused on demographic data and clinical information, such as comorbid medical conditions, colonization history and prior antibiotic exposures [3, 15–18, 29]. In the current study, we innovatively analysed microbiological parameters of colonizing strains to search for risk factors for subsequent infection after CRE colonization.

According to report of China Antimicrobial Surveillance Network (CHINET), CR-KP increased from 2.4 to 13.4% between 2005 and 2014 [30]. *K. pneumoniae* accounted for the largest percentage of CRE strains (66.7%) and 64% of *K. pneumoniae* isolates were ST11-KPC [31]. 85.7% of CRE strains were found to produce carbapenemases, among which KPC was predominant in *K. pneumoniae* isolates (77%) and NDM was predominant in *E. coli* isolates (75%) [31]. In accordance with domestic changing trend, CR-KP in our hospital increased significantly in recent two decades, for example, the detection rate of CR-KP in bloodstream infections was below 5% in 1998–2012 and increased to 34.9% in 2013–2017 [32]. ST11-KPC *K. pneumoniae* has caused a series of nosocomial outbreaks in China, including our hospital [33, 34]. There was an outbreak of CR-KP in the neonatal ward in 2015 in our hospital, among which ST11-KPC-2, ST20-NDM-1 and ST888-NDM-1 *K. pneumoniae* was 81.48% (22/27), 14.81% (4/27) and 3.70%(1/27), respectively [33]. Likewise, ST11-KPC *K. pneumoniae* was predominant in community-onset CRE (CO-CRE) infection. According to a tertiary hospital in China, *K. pneumoniae* accounted for 53.6% among 28 CO-CRE isolates, and 86.7% of *K. pneumoniae* strains belonged to ST11 containing *bla*_KPC-2_ [35]. Consistent with the prevalence of CRE strains from clinical specimens, *K. pneumoniae* was the most common rectal strain in our study, 51.9% of which were KPC-positive. Somewhat differently, approximately 30% of rectal strains were NCP-CRE; moreover, the proportions of KPC and NDM enzymes were approximately the same (31.5 and 35.2%, respectively). MLST analysis revealed a rich genetic diversity among intestinal *K. pneumoniae* strains, of which 36 distinct STs were identified, and ST11 was the most prevalent. The results of our study indicated that approximately all KPC-producing strains were ST11-*K. pneumoniae*, and half of NDM-producing strains were *E. coli*. Moreover, KPC-CRE had a high carbapenem MIC, and NCP-CRE had low imipenem and meropenem MICs.

Univariate analyses showed that there were differences in species, STs of *K. pneumoniae* strains, carbapenemase production status and carbapenemase-encoding genes between the two groups. Distributions of the above-mentioned microbiological parameters were concentrated in the infection group, in which KPC-2 *K. pneumoniae* was major strain type; by contrast, distributions were diverse and dispersed in the non-infection group, in which NCP-*K. pneumoniae* was the most prevalent*,* followed by NDM-5 *E. coli* and KPC-2 *K. pneumoniae*. After dichotomizing carbapenem MICs, the proportion of high-level carbapenem MICs was remarkably different between the two groups, in which the infection group was much higher. Among these significant variables, the OR of KPC-CRE was the highest, followed by high-level MICs of imipenem and ertapenem. Tamma P.D. et al. reported that ICU patients colonized with CP-CRE were more likely than NCP-CRE-colonized patients to develop CRE infections (36% vs. 5%) [19]. Our data showed that OR of CP-CRE was far less than that of KPC-CRE (3.993 vs. 13.028), suggesting that the correlation between CP-CRE colonization and subsequent infection was not as strong as KPC-CRE. In our previous study, carbapenem resistance score which based on the inhibition zone diameters of meropenem and imipenem was an independent risk factor for CRE bloodstream infection in intestinal carriers [36]. In line with previous report, high-level carbapenem MICs were remarkably relevant to subsequent infection in CRE-colonized patients in this study. It was noteworthy that carbapenem inhibition zone diameters of these CRE strains were usually 6 mm, hence the MICs value were more accurate. In addition, we analysed ertapenem resistance, which was shown to be a risk factor for subsequent infection.

We tried to improve the predictive ability by combining different indicators and found that they were not as good as KPC-CRE alone. Finally, we conducted multivariable analyses and found that only KPC-CRE or high-level imipenem MIC was an independent risk factor for infection when we included different significant variables. Our findings suggest that patients colonized with KPC-CRE or strains with imipenem MIC ≥ 32 mg/L may be at particularly high risk of subsequent CRE infections during their hospital stay. It is noteworthy that the precise epidemiology of carbapenemase is diverse across countries and regions. CR-KP strains harbouring KPC are prevalent in the United States, some parts of Europe and the Mediterranean region [37, 38], and most regions of China [31]; some countries are more affected by other carbapenemases, including Spain (VIM), India (NDM), most regions of the Middle East (except Israel) and north Africa (OXA-48) [38]; moreover, the NDM type is reported to be the key carbapenemase responsible for the carbapenem resistance phenotypes in children in some parts of China, including Shanghai [39, 40]. The predictive ability of KPC-CRE may not be generalizable to other hospitals and people.

Decolonization demonstrated a decline in CRE carriage rates and may be potentially useful for the prevention of a subsequent infection [10–12]. Common strategies for decolonization are selective digestive decontamination and faecal microbiota transplantation, which are promising but costly and invasive [10–13]. Various regimens for digestive decontamination have been investigated, including oral aminoglycosides (e.g. gentamicin), colistin and a combination of both [13]; however, it was reported that gut decontamination has been associated with the development of colistin and gentamicin resistance [41]. Our antibiotic susceptibility testing of rectal CRE strains showed a high susceptibility to colistin, and the susceptibility to gentamicin varied among species and carbapenemase types. Only 7.2% of strains producing KPC were susceptible to gentamicin, suggesting that phenotypic or genotypic testing of CRE colonizing strains is needed. Early identification of KPC-CRE-colonized patients is important because it may facilitate the targeted use of interventions and limit antimicrobial use.

This study also had several limitations. First, our study was conducted in the department of haematology and ICU, and the prevalence of CRE colonizing strains and risk factors may not be generalizable to other institutions or departments. Second, we did not confirm that the colonizing and clinical infection CRE isolates were the same. It is of great clinical significance to match the CRE colonization isolate and subsequent infected isolate on all available microbial parameters, including bacterial species, phenotypic antimicrobial resistance profile, antimicrobial resistance genes, virulence genes, etc., which is the theoretical basis for secondary infection caused by CRE colonization. In the follow-up study, we will analyse the homology of CRE colonization strains and infection strains by multiple technique, such as pulsed field gel electrophoresis (PFGE) and whole-genome sequencing (WGS). Moreover, we performed MLST of *K. pneumoniae* isolates only, MLST analyses of other species were not evaluated. In the future, we will accumulate more cases and analyse the association between MLST stratified by bacterial species and subsequent CRE infection. Finally, patients’ clinical information, which was associated with a subsequent infection to a certain extent, was not included. Combined analysis of bacterial and clinical factors may improve the predictive ability.

## Conclusions

In summary, this was an innovatively study to investigate colonizing isolates on all available microbiological parameters, expanding our understanding of the crucial factors of colonizing strains in the incidence of a CRE infection. Our findings suggest that phenotypic or genotypic testing of colonizing CRE strains is needed, and patients colonized with KPC-CRE or strains with imipenem MIC ≥ 32 mg/L are more likely to develop subsequent CRE infections during their hospital stay.

## Supplementary Information

**Additional file 1.**

## Data Availability

The datasets used and/or analysed during the current study are available from the corresponding author on reasonable request.

## Abbreviations

CRE: Carbapenem-resistant *Enterobacteriaceae*
ICU: Intensive care unit
MLST: Multilocus sequence typing
KPC: *K. pneumoniae* carbapenemase
NCP-CRE: Non-carbapenemase-producing CRE
NDM: New Delhi metallo-β-lactamase
MIC: Minimum inhibitory concentration
aOR: Adjusted odds ratio
CI: Confidence interval
CDC: Centers for Disease Control and Prevention
CP-CRE: Carbapenemase-producing CRE
MALDI-TOF MS: Matrix-assisted laser desorption/ionization time-of-flight mass spectrometry
CLSI: Clinical and Laboratory Standards Institute
EUCAST: European Committee on Antimicrobial Susceptibility testing
mCIM: Modified carbapenem inactivation method
PCR: Polymerase chain reaction
ST: sequence types
OR: Odds ratio
CR-KP: Carbapenem-resistant *K. pneumoniae*
