# Supplementary material for: Bacterial characteristics of carbapenem-resistant Enterobacteriaceae (CRE) colonized strains and their correlation with subsequent infection
Source: BMC Infect Dis. 2021 Jul 2;21:638. doi: 10.1186/s12879-021-06315-0 (PMC8254368; doi:10.1186/s12879-021-06315-0)
Supplement: Supplementary file 1 — Additional file 1. [file 12879_2021_6315_MOESM1_ESM.docx]

Supplementary Table 1. Carbapenem-resistant *Enterobacteriaceae* (CRE) colonization strains in different groups

|  | **Total No.** | **Infection group(n=58)** | | | **Non-infection group(n=161)** | | |
| --- | --- | --- | --- | --- | --- | --- | --- |
| **Organism** |  | **No.** | **Carbapenemase** | **ST** | **No.** | **Carbapenemase** | **ST** |
| *Citrobacter amalonaticus* | 1 | 0 | / | / | 1 | NDM-1(1) | / |
| *Citrobacter freundii* | 11 | 0 | / | / | 11 | NDM-1(8), NDM-7(1), NDM-5(1), -(1) | / |
| *Enterobacter cloacae* | 10 | 2 | NDM-1(2) | / | 8 | NDM-1(6), +(1), -(1) | / |
| *Enterobacter kobei* | 4 | 1 | NDM-1(1) | / | 3 | NDM-1(3) | / |
| *Escherichia coli* | 54 | 7 | NDM-5(4), NDM-1(1), -(2) | / | 47 | KPC-2(1), NDM-1(4), NDM-4(1), NDM-5(26), VIM-1(2), -(13) | / |
| *Klebsiella oxytoca* | 2 | 0 | / | / | 2 | NDM-1(1), KPC-2&NDM-1(1) | / |
| *Klebsiella pneumoniae* | 133 | 47 |  |  | 86 | KPC-2(26) | ST11(25), ST15(1) |
|  |  |  |  |  |  | IMP-4(1) | ST899(1) |
|  |  |  | KPC-2(42) | ST11(41), ST1(1) |  | KPC-2&NDM-1(1) | ST (1) |
|  |  |  | NDM-5(1) | ST3387 |  | NDM-1(6) | ST2264(2), ST4290(1), etc^a^ |
|  |  |  | -(4) | ST469(1), ST37(1), ST15(1), ST11(1) |  | NDM-5(8) | ST43(3), ST2243(1), etc^b^ |
|  |  |  |  |  |  | NDM-7(1) | ST485(1) |
|  |  |  |  |  |  | +(1) | ST2941(1) |
|  |  |  |  |  |  | -(42) | ST37(10), ST15(6), ST147(5), ST307(3), c |
| *Morganella morganii* | 1 | 1 | -(1) | / | 0 | / | / |
| *Raoultella ornithinolytica* | 2 | 0 | / | / | 2 | IMP-4(1), OXA-48(1) | / |
| *Leclercia adecarboxylata* | 1 | 0 | / | / | 1 | NDM-1(1) | / |

Note:

a.ST3884(1), ST1035(1), ST147(1)

b.ST1803(1), ST617(1), ST483(1), ST35(1)

c.ST22(2),ST2133(2),ST1128(1),ST1119(1),ST894(1),ST656(1),ST632(1),ST540(1),ST452(1),ST394(1),ST234(1),ST194(1),ST40(1),ST4267(1),ST2407(1)

Supplementary Table 2. The statistical evaluation index at each possible cutoff value for the dichotomized minimum inhibitory concentration (MIC) of carbapenem

| **Value(mg/L)** | **Sensitivity** | **Specificity** | **Youden index** | **PPV** | **NPV** | **Accuracy rate** | **Misdiagnosis rate** | **Omission di-agnostic rate** |
| --- | --- | --- | --- | --- | --- | --- | --- | --- |
| MIC of meropenem | | |  |  |  |  |  |  |
| ≥8 | 87.9 | 30.4 | 18.3 | 31.3 | 87.5 | 45.7 | 69.6 | 12.1 |
| ≥16 | 87.9 | 37.9 | 25.8 | 33.8 | 89.7 | 51.2 | 62.1 | 12.1 |
| ≥32 | 84.5 | 48.4 | 32.9 | 37.1 | 89.7 | 58.0 | 51.6 | 15.5 |
| ≥64 | 81.0 | 64.6 | 45.6 | 45.2 | 90.4 | 69.0 | 35.4 | 19.0 |
| ≥128 | 60.3 | 83.2 | 43.5 | 56.5 | 85.4 | 77.2 | 16.8 | 39.7 |
| MIC of imipenem | | |  |  |  |  |  |  |
| ≥8 | 87.9 | 42.2 | 30.1 | 35.4 | 90.7 | 54.4 | 57.8 | 12.1 |
| ≥16 | 81.0 | 64.6 | 45.6 | 45.2 | 90.4 | 69.0 | 35.4 | 19.0 |
| ≥32 | 75.9 | 79.5 | 55.4 | 57.1 | 90.1 | 78.5 | 20.5 | 24.1 |
| ≥64 | 25.9 | 95.0 | 20.9 | 65.2 | 78.1 | 76.7 | 5.0 | 74.1 |
| ≥128 | 5.2 | 98.8 | 4.0 | 60.0 | 74.3 | 74.0 | 1.2 | 94.8 |
| MIC of ertapenem | | |  |  |  |  |  |  |
| ≥8 | 96.6 | 14.9 | 11.5 | 29.0 | 92.3 | 36.6 | 85.1 | 3.4 |
| ≥16 | 86.2 | 28.0 | 14.2 | 30.1 | 84.9 | 43.3 | 72.0 | 13.8 |
| ≥32 | 82.8 | 54.7 | 37.5 | 39.7 | 89.8 | 62.1 | 45.3 | 17.2 |
| ≥64 | 75.9 | 73.9 | 49.8 | 51.2 | 89.5 | 74.4 | 26.1 | 24.1 |
| ≥128 | 51.7 | 84.5 | 36.2 | 54.5 | 82.9 | 75.8 | 15.5 | 48.3 |

Abbreviations: MIC, minimum inhibitory concentration; PPV, positive predictive value; NPV, negative predictive value.

Supplementary Table 3. Multivariable analyses of bacterial factors for subsequent infection among patients with carbapenem-resistant *Enterobacteriaceae* (CRE) colonization

|  | **Dichotomizing by KPC producing** | | **Dichotomizing by carbapenemase producing** | |
| --- | --- | --- | --- | --- |
|  | **aOR (95% CI)** | ***P*** | **aOR (95% CI)** | ***P*** |
| Species | 0.827(0.296-2.312) | 0.718 | 1.365(0.536-3.472) | 0.514 |
| MIC of meropenem | 1.292(0.280-5.965) | 0.743 | 1.481(0.271-8.079) | 0.650 |
| MIC of imipenem | 5.348(0.851-33.631) | 0.074 | 9.515(1.617-55.977) | 0.013 |
| MIC of ertapenem | 0.616(0.087-4.348) | 0.627 | 0.908(0.148-5.569) | 0.917 |
| Carbapenemase | 4.507(1.339-15.171) | 0.015 | 0.809(0.205-3.190) | 0.762 |

Abbreviations: MIC, minimum inhibitory concentration; KPC, *K. pneumoniae* carbapenemase; aOR, adjusted odds ratio; CI, confidence interval.

**
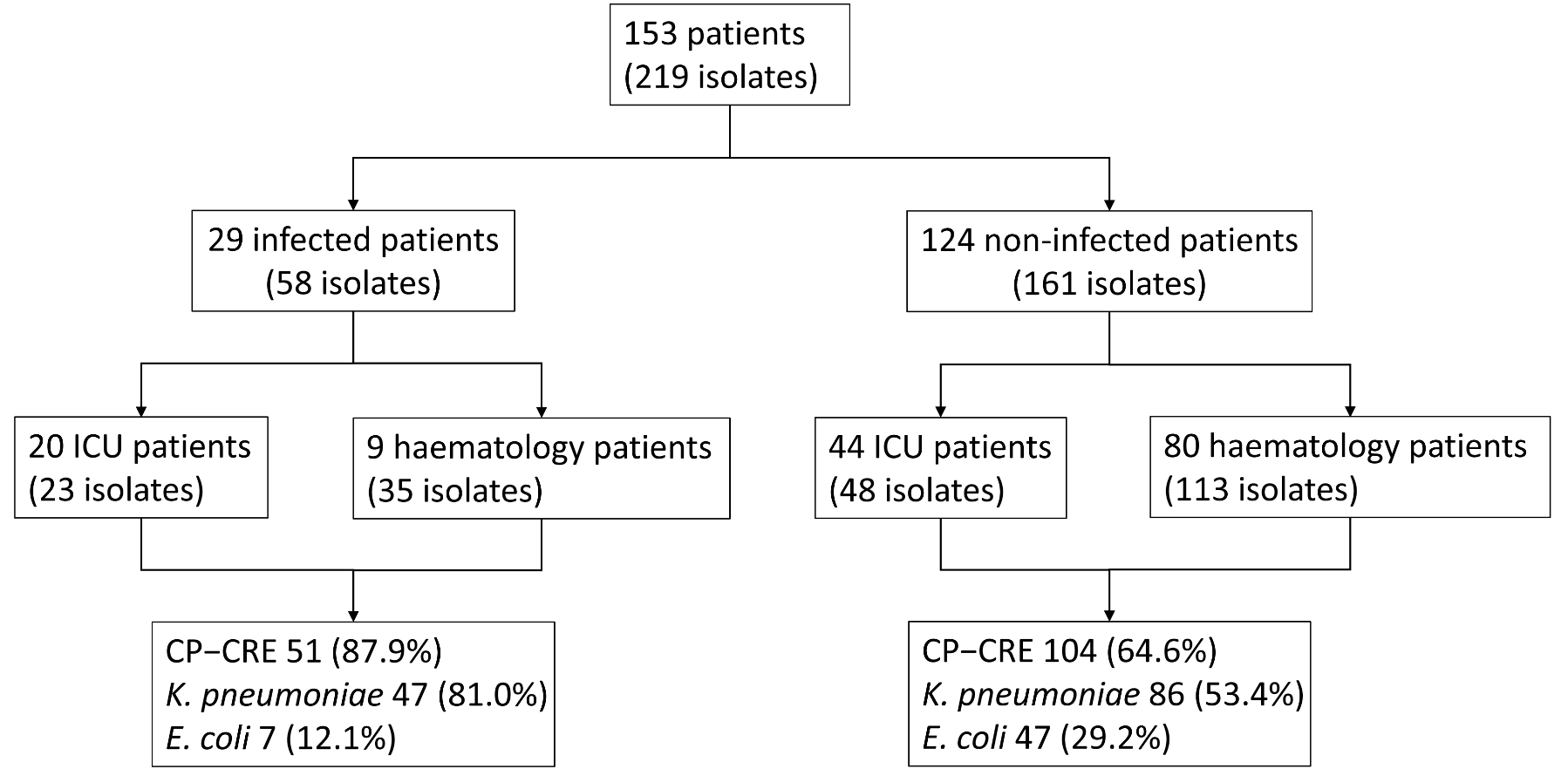
**

Supplementary Figure 1. The distribution of included carbapenem-resistant *Enterobacteriaceae* (CRE)-colonizing strains. ICU, intensive care unit; CP-CRE, carbapenemase-producing CRE.
